# Silicosis in a Countertop Fabricator — Texas, 2014

**Published:** 2015-02-13

**Authors:** Gary K. Friedman, Robert Harrison, Heidi Bojes, Karen Worthington, Margaret Filios

**Affiliations:** 1 Pulmonary Division, University of Texas Health Houston; 2 California Department of Public Health; 3 Texas Department of State Health Services; 4 New Jersey Department of Health and Senior Services; 5 Division of Respiratory Disease Studies, National Institute for Occupational Safety and Health, CDC

In May 2014, the Texas Department of State Health Services was notified of a case of silicosis with progressive massive fibrosis in a Hispanic male aged 37 years who worked for an engineered stone countertop company as a polisher, laminator, and fabricator. He was exposed to dust for 10 years from working with conglomerate or quartz surfacing materials containing 70%–90% crystalline silica.* This is the first reported case of silicosis associated with exposure to quartz surfacing materials in North America.

In 2010, the patient presented to a primary care provider with a 2-year history of persistent cough and dyspnea on exertion. He had no history of tobacco use or pulmonary disease. On physical examination, he had diminished bibasilar breath sounds and a right-sided inspiratory wheeze. Pulmonary function studies showed a combined obstructive and restrictive defect with no change post bronchodilator and reduced diffusion capacity. An electrocardiogram showed right ventricular hypertrophy, and cardiac catheterization confirmed the presence of pulmonary hypertension. A B Reader† classified the patient’s chest radiograph as large opacity Category “C” with 3/2 profusion, q/r bilateral upper and middle lobe rounded opacities. Computed tomography scan of the chest showed bilateral upper and middle lobe small rounded and large opacities, with hilar and mediastinal adenopathy. The worker was reassigned to a different job to minimize silica dust exposure. He is oxygen-dependent, and his medical condition is being monitored for possible lung transplantation.

Clusters of silicosis cases, some requiring lung transplantation, have occurred among fabrication workers exposed to silica dust from quartz surfacing materials in Israel, Italy, and Spain (1–4). In the last year, imports of quartz surfacing materials to the United States have risen 49%,§ and these materials are among the most popular countertop materials. The increased use of this silica-containing material poses a new risk for silica exposure (http://blogs.cdc.gov/niosh-science-blog/2014/03/11/countertops). An investigation by CDC’s National Institute for Occupational Safety and Health of the patient’s work site is ongoing to identify work hazards and assess silica exposures and the health of the other employees.

Health care providers need to be aware of quartz surfacing materials as a source of silica exposure, advise reassignment of patients with silicosis to jobs without silica dust exposure, and report cases to their state public health agency; in 2010, silicosis was reportable in 25 states.¶ Employers are responsible for maintaining a safe workplace by measuring silica exposure, limiting access to areas where silica exposures are high, using effective methods to reduce exposure (e.g., wet methods,** local exhaust ventilation, and use of personal protective equipment), providing medical examinations to workers with high exposures, and training workers about silica hazards and how to limit exposures.††

## Footnotes

* Additional information available at http://www.4willis.com/pdf/zodiaq/msds-zodiaqslab_aug2010.pdf, http://www.caesarstoneus.com/en/pages/tech%20-%20info.aspx, and http://www.silestoneusa.com/distributors/for-the-trade.

† Additional information available at http://www.cdc.gov/niosh/topics/chestradiography/breader.html and http://www.cdc.gov/niosh/topics/chestradiography/ilo.html.

§ Information available at http://www.stoneupdate.com/us-stone-imports/statwatch-monthly-report/805-granite-quartz-imports-continue-2014-boom?utm_source=mailinglist&utm_medium=email&utm_campaign=edge+16+december+2014.

¶ In 2010, silicosis was a reportable condition in 25 states (Arkansas, California, Connecticut, Delaware, Florida, Illinois, Iowa, Kentucky, Maine, Maryland, Massachusetts, Michigan, Minnesota, Missouri, Nevada, New Hampshire, New Jersey, New Mexico, New York, North Carolina, Ohio, Rhode Island, Texas, Virginia, and Wisconsin); however, only two states (Michigan and New Jersey) currently submit case data to CDC’s National Institute for Occupational Safety and Health. Additional information available at http://www.cste.org/group/srcaqueryres.

** Suppression of dust using water stream or spray.

†† Additional information available at https://www.osha.gov/dsg/topics/silicacrystalline/index.html, https://www.osha.gov/OshDoc/data_General_Facts/crystalline-factsheet.pdf.
